# A new preclinical-year revision course designed and taught by clinical-year students: analysis of tutees' perceived confidence in an innovative teaching model

**DOI:** 10.1186/1753-6561-6-S4-O50

**Published:** 2012-07-09

**Authors:** M Protty, J Mann, M Mohammed, C Wiskin

**Affiliations:** 1 College of Medical and Dental Sciences, University of Birmingham, UK

## Introduction

The GMC has emphasized the importance of teaching skills for doctors and recommended that training for these skills is introduced early on in the medical education curriculum. One way to achieve this is in the form of near-peer assisted learning, where junior medical students are taught by senior ones. We here report the development of an extensive near-peer teaching project at the University of Birmingham and its impact on tutees. To evaluate the impact this near-peer teaching project has had on the perception of junior medical students of their confidence in their knowledge of the topics covered.

## Methods

The course was fully designed and delivered by three medical students in the clinical years and consisted of 76 hours of teaching in total, split equally between year 1 and 2 preclinical topics. Tutees were asked to complete evaluation forms before and after each session, answering a range of questions that included general demographic details as well as a self-rating of confidence in the topic taught before and after on a 100mm visual analogue scale (VAS).

## Results

The average overall student rating of their perceived self-confidence in the topics before the sessions was 42.6. All sessions individually showed significant increases in confidence ratings after the sessions, with an average rating of 61.7 (p<0.01).

## Conclusions

The near-peer teaching model was widely accepted by the tutees and significantly improved the confidence in the topics taught. Further studies need to be done to determine the value of such models in core medical curricula.

